# The effect of refining process on the physicochemical properties and micronutrients of rapeseed oils

**DOI:** 10.1371/journal.pone.0212879

**Published:** 2019-03-08

**Authors:** Ying Wu, Runsong Zhou, Zhigao Wang, Bo Wang, Yijie Yang, Xingrong Ju, Rong He

**Affiliations:** 1 College of Food Science and Engineering, Collaborative Innovation Center for Modern Grain Circulation and Safety, Key Laboratory of Grains and Oils Quality Control and Processing, Nanjing University of Finance and Economics, Nanjing, China; 2 National Engineering Laboratory for Cereal Fermentation Technology, School of Food Science and Technology, Jiangnan University, Wuxi, China; University of Hawai’i at Manoa, UNITED STATES

## Abstract

Information on the physicochemical variability in rapeseed oil from different varieties during each refining process is lacking. Our purpose was to investigate the physicochemical properties, micronutrients and oxidative stability of the oil extracted from the five varieties of rapeseeds during their different stages of refining process. Increase in the acid value, peroxide value and *p*-anisidine value were detected in the refining, while content of tocopherols, sterols, β-carotene and phenols, which are regarded as important nutritional compounds diminished. Moreover, the loss rate of total phytosterols of all oils during neutralization (9.23–7.3%) and deodorization (9.97–8.27%) were higher than that of degumming (3.01–0.87%) and bleaching (2.75–1.18%). Deodorization affected total tocopherols contents the most, followed by bleaching, neutralization and degumming. There was a remarkable reduction in total content of phenol, β-carotene and oxygen radical absorbance of all oils during refining. The accumulated information can be used in looking for the optimum condition to meet the basic requirements for oil and minimize micronutrients losses so as to increase their market value.

## Introduction

Rapeseed oil is a major source of vegetable oil in the world. Its production is estimated at 27.71 million metric tons, the third just behind palm and soybean oil, supporting the 2018 official data of United States Department of Agriculture. Rapeseed oil contains around 1.5–6% palmitic acid, around 0.5–3.1% stearic acid, around 28% oleic acid, around 11–23% linoleic acid, and around 5–13% linolenic acid. It is characterized by the ideal content of polyunsaturated fatty acid due to the ratio (2:1) of linoleic acid (n-6) and linolenic acid (n-3) fatty acid. On the other hand, the rapeseed oil contains many active biological compounds such as phytosterols, tocopherol, phenolic compounds, β-carotene [1]. Quality and stability are the main factors in the oil production which decide acceptance and sales of vegetable oil products [2,3]. However, the fatty acid composition and minor components such as free fatty acids, phospholipids, trace metals, color pigments, phenolic compounds and waxes of vegetable oil products often affect quality and stability [1,4].

Phosphatides, free fatty acids, odiferous volatiles, colourant, waxes and metal compounds, which are undesirable materials in crude rapeseed oil products, have adverse effects on physicochemical properties, sensory characteristics, and storage stability of the rapeseed oil. Towards this, the crude rapeseed oil needs refining treatments to remove undesirable ingredients and produce higher quality oils [5–7]. Traditional rapeseed oil refining processes generally involve four successive operations: neutralization, degumming, bleaching and deodorization. Although the traditional chemical refining processes are useful to remove the majority of these unwanted components and could achieve the desired color and a mild taste, micronutrients such as tocopherol, phytosterols, β-carotene and phenolic compounds will have at somewhat loss during the refining process, and antioxidant activity of oil will change as well. Moreover the oxidation of lipid and its associated substances, lipid degradation products, toxic and harmful substances will exist in the oil products during the refining process and then affect the quality and function of products [8].

In the last decades, many researchers have focused on the evaluation of influence of different compositional parameters on quality and stability of vegetable oils. For instance, Klara Kraljic´ explored changes in phenolic compounds during specific refining process of rapeseed oil [9]. Aleksandra Szydłowska-Czerniak studied the relation between loss of bioactive compounds and antioxidant capacity in the refining process of palm oil [10]. Jia Wei optimized refining method for the most suitable temperatures to retain several essential fatty acids and bioactive compounds in the tea seed oil [11]. Although many researchers have investigated the physicochemical properties of rapeseed oil, there is still a lack of information on the physicochemical properties and oxidative stability as it relates to different rapeseed varieties during refining process.

The need for a more comprehensive study for the physicochemical properties and oxidative stability of rapeseed varieties available in the marketplace is crucial. Thus, the first objective of this work was to investigate the physicochemical properties of five different kinds of rapeseed oils during different stages of traditional refining process; secondly, the study aims to broaden knowledge and provide helpful data for the future optimization of the traditional oil refining process without affecting the desirable compounds.

## Materials and methods

### Materials

Tocopherol standards (α-tocopherol, β-tocopherol, γ-tocopherol and δ-tocopherol, purity>98%) were obtained from Sigma-Aldrich Chemical (Nanjing, China). Brassicasterol, campesterol, and β-sitosterol standards (purity≥98%) were also purchased from Sigma-Aldrich Chemical (Nanjing, China). N-hexane, methanol, anhydrous ethanol, acetonitrile (HPLC grade) were obtained from Merck (Nanjing, China). Trolox, AAPH, fluorescein, PBS and other solvents and reagents (analytical grade) were purchased from Sinopharm Chemical Reagent (Shanghai, China).

### Preparation of oil samples

Commercial rapeseeds (*Brassica napus* L.) were obtained from the local market. They were categorized into five varieties, namely Zhongshuang 11, Fengyou 5103, Deyou 8, Zhongyou 6766 and Huyou 4. Pressing was adopted after roasting (125°C, 30min) for crude oil production. Refining processes in the laboratory was carried out by using operating conditions as described before with slight modifications [12]. Water degumming with acid and neutralization using sodium hydroxide were performed. Afterwards, the soap stock of the neutralized oil was removed from the rapeseed oil after centrifugation at 6000g for 10 min. The oil was bleached using bleaching earth, and was then deodorized at four temperatures: 190°C, 210°C, 230°C, 250°C and 270°C under vacuum by the injection of steam. Table 1 showed the summary of the conditions used in the different stages of the refining process.

**Table 1:** Summary table of different conditions used in different stages of the refining process.

Stage	Temperature(°C)	Time(min)	Materials and chemicals used
Degumming	55	30	Phosphoric acid (0.2% w/w) Ultra-pure water (4% w/w)
Neutralization	65	30	NaOH solution (16° Baume)
Bleaching	95	20	Acid-activated bleaching earth (3% w/w)
Deodorization	250	80	-

### Determination of physicochemical properties

Acid value (AV) was measured according to *GB*.*5009*.*229–2016* and expressed as mg KOH/g oil. Peroide value (PV) was evaluated by *GB*.*5009*.*227–2016* and expressed as mmol/kg oil. *p*-Anisidine value (*p*-AV) was determined following *GB/T 24304–2009*.

### Determination of color

CIE L*, a*, b* color. Color was measured in the mode of liquid measurement using KONICA MINOLTA CM-5 colorimeter with the EasyMatch QC software according to preivoous study [12].

### Determination of phytosterols

The content of phytosterol was analyzed according to previous procedure with slight modifications [13]. A 200 mg of rapeseed oil was mixed with 4mL potassium hydroxide ethanol solution (2mol/L) and shaken in a water bath at 90°C for 1h for saponification. When samples were cooled down to room temperature, 5 mL of n-hexane and 1 mL of deionized water were added and mixed vigoursly to extract the unsaponifiable matter. The supernatant was pooled again by the same procedure. Extraction solution was blown dry by nitrogen. 200 μL BSTFA-TMCS(99:1) was added into sample, and then the sample was shaken in a water bath at 99°C for 20min for derivatization. At last the sample was diluted to 2 mL by adding n-hexane for detection. Gas Chromatography Tandem Mass Spectrometry (GC-MS) (Agilent Technologies 7890B-7000C, USA), equipped with a HP-5MS column (30 m × 0.25 mm i.d., 0.25 μm film thickness; Agilent Technologies). The split ratio was 30:1. The carrier gas was helium at a constant flow rate of 1.0 mL/min. The temperatures of injector, transfer line, ion source, and quadrupole mass filter were set at 270, 280, 230, and 150°C, respectively. The oven temperature was programmed from 180°Cto 280°C at 10°C/min and held for 15 min. The solvent delay time was 3 min. The electron ionization voltage was 70 eV and the ion scan range was 50–500 amu. The concentration of each phytosterol was expressed as μg/g oil.

### Determination of tocopherols

Content of tocopherols was measured according to a previous procedure [14], with some modifications. Briefly, 0.2g of each samples were dissolved into 5 mL methanol. After centrifugation for 10 min and the supernatant (10 μL) was injected into the HPLC system (Agilent Technologies1260, USA) equipped with a ultraviolet detector on a C18 column (250mm×4.6mm, i.d., 5μm film thickness; Agilent Technologies). A mixture of acetonitrile-water (90:10 v/v) at a flow-rate of 1.0 ml/min (isocratically) was used as a mobile phase. Absorbance was measured at λ_292_ nm. Different tocopherol species were identified by comparing their retention times to those of α-, β-, γ- and δ-tocopherol mixed standard. An external standard method was employed for quantification and the content was expressed as μg/g oil.

### Determination of total phenols content (TPC)

The TPC content was determined spectrophotometrically at 765 nm using the Folin-Ciocalteu reagent, as described before [15], The TPC results of the samples was expressed as mg of GAE (gallic acid equivalents)/ 100g oil.

### Determination of β-carotene content

To isolate and quantify β-carotene, we used the procedure described by Gimeno [16], and made some modifications. 200 mg of rapeseed oil and 4 mL potassium hydroxide ethanol solution (2mol/L) were accurately added into a 50 mL centrifuge tube. Then the centrifuge tube was shaken at 80°C for 1h for saponification. After the samples cooled down, 5 mL of n-hexane and 1 mL of deionized water were added to 50mL centrifuge tube to extract the unsaponifiable matter (spiral shock for 2 min and then centrifuged to obtain the supernatant). The organic phases obtained were evaporated to the dry fraction using a rotavaporat at 40 °C. The residue was brought to dryness with nitrogen and then re-dissolved in methanol. In this process, the chromatographic injection must be performed as fast as possible to avoid the oxidation and decomposition of the β-carotene. The β-carotene content of the samples was expressed as mg/ 100g oil.

### Determination of oxygen radical absorbance capacity

The ORAC assay was performed according to previous procedure [17], with minor modifications. The reaction was performed in 75 mM phosphate buffer (pH 7.4), and the final assay volume was 200 μL. Briefly, 20 μL of the sample (Methanol extraction of oil) and 120 μL of a 70 nM fluorescein solution (final concentration of 0.96 μM) were placed in 96-well plates. The mixture was held at 37 °C for 20 min. Next, 20 μL of 119 mM AAPH was added to each well. The fluorescence intensity was measured using a SpectraMax M2e microplate reader at an excitation of 485 nm and an emission of 538 nm for 35 cycles at 5 min intervals. The area under the curve (AUC) was calculated using SoftMax Pro software (version 5.4.1) from which the blank was then subtracted to obtain the net AUC. The linear regression equation for the net AUC versus the concentration of RAP was calculated. The final ORAC values were expressed as μmol TE (Trolox equivalent)/100g oil.

### Determination of fatty acid composition

Fatty acid composition was determined using the method in a previous study with slight modifications [18]. A 50 mg of oil was dissolved in 2 mL of 3M methanolic potassium hydroxide. The mixture was then vigorously vibrated for 180s and placed into a water bath at 50°C for 30min. After conversion of fatty acid methyl ester was completed, 5 mL hexane was added into mixture. The mixture was vigorously vibrated for 180s and allowed to stand for 5min for the upper layer to become clear. The supernatant was purified using a 0.22-μm filter and then analyzed on an Gas Chromatography Tandem Mass Spectrometry (GC-MS) (Agilent Technologies 7890B-7000C, USA), equipped with a HP-innowax column (30m× 0.25 mm i.d., 0.25 μm film thickness; Agilent Technologies) with a split ratio of 10:1. The carrier gas was helium at a constant flow rate of 2.0 mL/min. The temperatures of injector, transfer line, ion source, and quadrupole mass filter were set at 260, 280, 200, and 150°C, respectively. The oven temperature program of the DB-5 column was held at 140°C for 5 min and was then increased to 240°C at a rate of 4°C/min, where it was held for 30 min. The solvent delay time was 5 min. The electron ionization voltage was 70 eV and the ion scan range was 50–550 amu. The fatty acid methyl esters (FAME) were analyzed in GC-MS for identification of different components.

The deodorized time was set at 80min and the deodorized temperature was set at 190°C, 210°C, 230°C, 250°C and 270°C. Percentage content of trans-linoleic and trans-linolenic were determined to study the effect of temperature on trans fatty acids.

### Statistical analysis

All the samples were analyzed in duplicate. Statistical analysis was performed using SPSS (version 19. 0) and origin (version 8. 5). Differences were considered statistically significant at P< 0.05.

## Results and discussion

### Analyses of physiochemical properties of rapeseed oils

**Fig 1:**
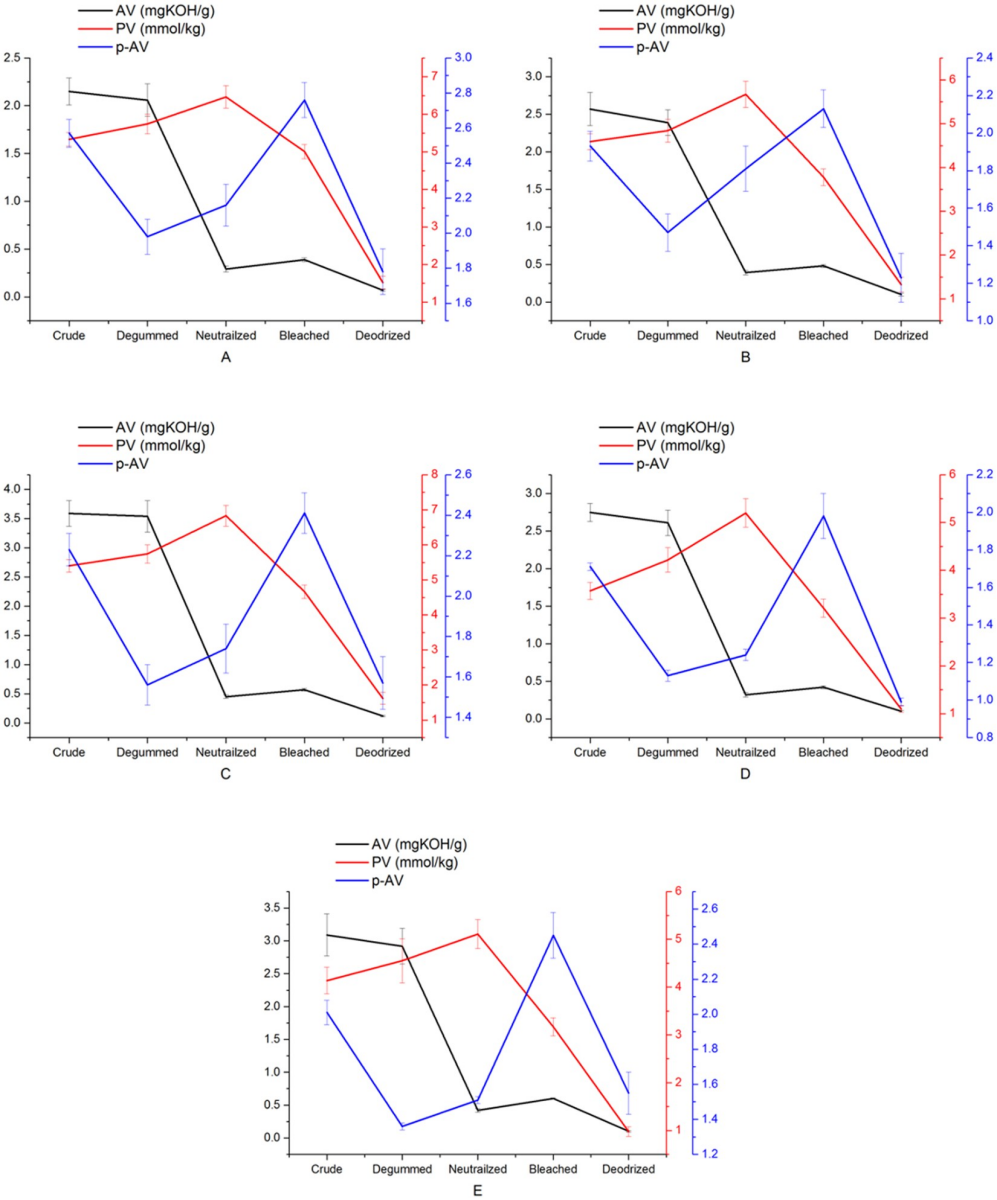
Acid value, peroide value, and p-Anisidine value of oils of number 1 (A), number 2 (B), number 3 (C), number 4 (D), number 5 (E) during every refining step including the degumming, neutralization, bleaching and deodorization.

The AV, PV, *p*-AV of the rapeseed oil in varieties after each refining process are shown in Fig 1. The AV of five different kinds of crude rapeseed oils were from 2.15 to 3.59 mg KOH/g and decreased significantly after neutralization, ranging from 0.29 to 0.45 mg KOH/g. The AVs significantly decreased after neutralization, probably due to the adding of NaOH that led to the formation of sodium soaps in oil [19,20]. However, the slight increase (0.29–0.45 to 0.39–0.60 mg KOH/g) of the AV of five different kinds of neutralized rapeseed oils during bleaching was observed probably due to the ester hydrolysis caused by the bleaching earth. Then, the deodorization decreased the AV of all kinds of rapeseed oils again. According to previous study, this decrease was due to the volatilization of FFA as deodorization was a process of vacuum-steam distillation [21]. The level of AV indicates degree of hydrolytic rancidity of the oil [22]. The low AV of refined rapeseed oil would make rapeseed oil edible and the odor of the refined oil without offensive smell.

Peroxide value reflects the formation of hydroperoxides caused by the primary oxidation. An increase in PV of oil is an indicator of the oxidative deterioration [23]. As illustrated in Fig 1, degumming-neutralization increased the PV significantly, while bleaching and deodorization decreased the PV of all kinds of rapeseed oils significantly. The increase of PV of all kinds of crude rapeseed oils (3.57–5.40 to 5.11–6.83 mmol/kg) during degumming-neutralization could be attributed to the generation of the primary oxidation products caused by lipoxygenases, air, heat, moisture, and light activities [21]. Furthermore, a significant decrease of PV of bleaching oils in varieties (5.11–6.83 to 3.17–5.01 mmol/kg) after neutralization can be attributed to the absorbance and catalysis of peroxide compounds by bleaching earth. Finally, deodorization significantly decreased the PV of all kinds of bleached rapeseed oils, which might be attributed to the volatilization and further decomposition of peroxides caused by the high temperature. Previous study [24] indicated that the PV of oils was closely related to the operational parameters of deodorization, such as temperature, heating time, and steam rate.

*p*-AV indicates the presence of secondary oxidation products of lipids. Fig 1 showed the whole refining process decreased the *p*-AV of five different kinds of crude rapeseed oils significantly (2.57–1.71, before refining; 1.78–0.99, after refining). It was noteworthy that *p*-AV of five kinds of crude rapeseed oils were significantly reduced during degumming step (from 2.57–1.71 to 1.98–1.13), whereas neutralization and bleaching step led to significant increase of *p*-AV of all kinds of rapeseed oils (from 1.98–1.13 to 2.76–1.98), which could be owing to decomposition of the hydroperoxides and formation of secondary oxidation products, such as aldehydes and ketones because of catalytic properties of acid-activated bleaching earth.

**Fig 2:**
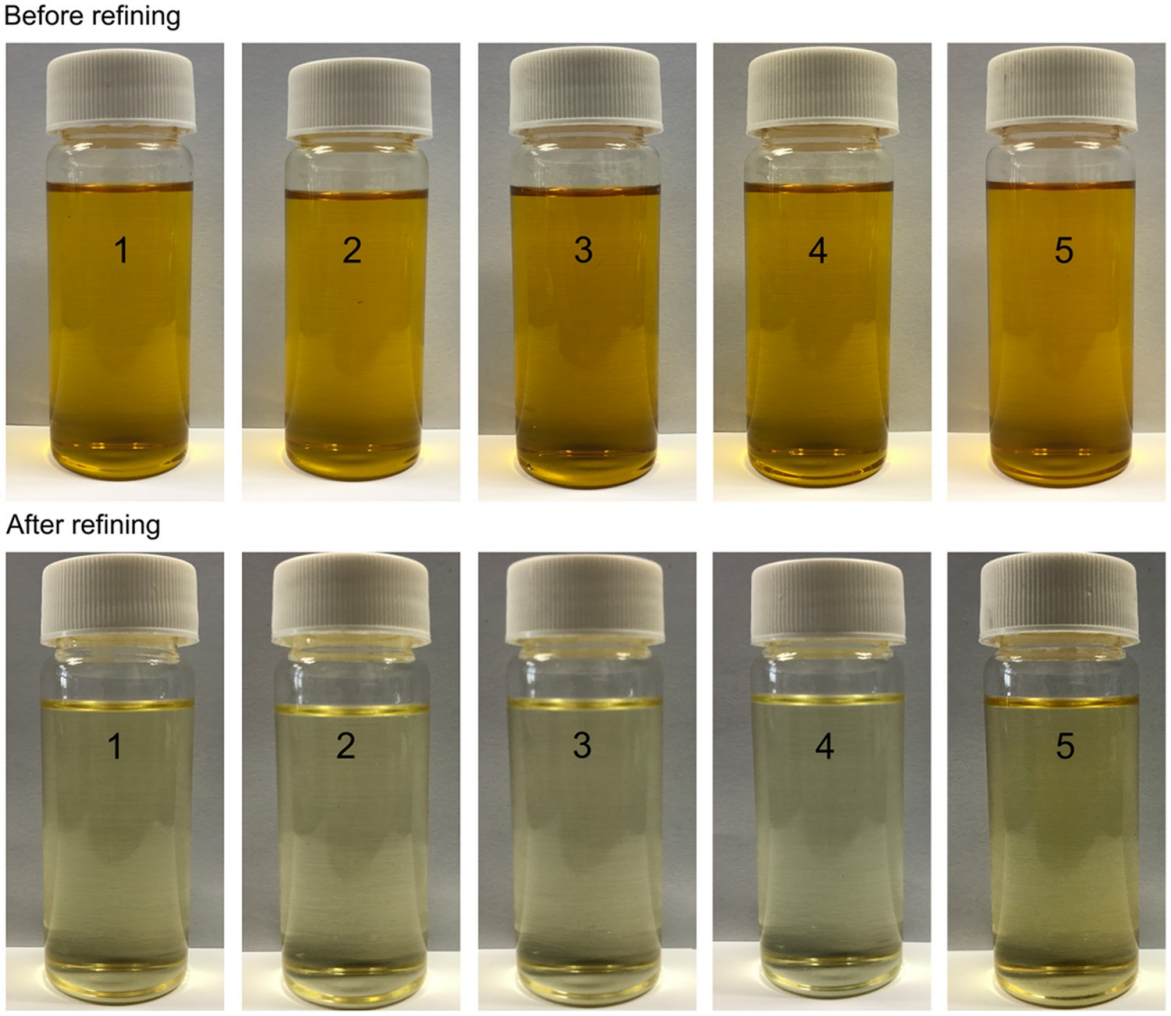
Samples of oils of number 1 (A), number 2 (B), number 3 (C), number 4 (D), number 5 (E) before and after refining.

Color is one of the physicochemical properties that represents the level of oil refining, which can affect consumer choice at somewhat. Carotenoids and chlorophyll are the main color pigments in vegetable oils. Oxidation of these compounds or reaction of oxidized triglycerides with carotenoids could cause oil darkening [25]. As shown in Table 2 and Fig 2, the lightness increased from 90.35–91.58 to 99.54–102.19, the redness decreased from 2.77–5.48 to 2.46–4.93 and yellowness decreased from 126.49–133.93 to 6.95–18.25 in the whole refining process. However, the yellowness of oils of Huyou 4 (18.25) and Zhongshang 11 (15.69) after deodorization were higher than that of other 3 kinds oils (9.16–6.95). This could be attributed to the different varieties of seeds. Bleaching is the most important step for removing color because of activated bleaching earth [20]. In current research, the color of all oils could be removed completely after deodorization, probably owing to degradation of highly unsaturated carotenoids during deodorization.

**Table 2:** Color of five different kinds of rapeseed oils before and after refining.

Varieties	Rapeseed oil before refining			Rapeseed oil after refining		
	L*	a*	b*	L*	a*	b*
1	90.43±2.34a	2.77±0.23a	126.49±3.45a	99.54±2.12b	-2.88±0.21b	15.69±1.11b
2	91.18±3.34a	3.78±0.49a	131.49±4.21a	101.01±3.19b	-4.49±0.17b	9.16±0.78b
3	90.35±2.15a	5.48±0.45a	133.93±2.12a	101.15±1.90b	-4.93±0.31b	8.67±0.86b
4	91.58±4.21a	3.06±0.56a	130.46±3.78a	102.00±3.45b	-2.49±0.22b	6.95±0.67b
5	91.41±1.12a	3.12±0.32a	130.88±5.67a	102.19±2.78b	-2.46±0.37b	18.25±2.12b

L*, lightness, range: −180 (black) to +180 (white); a*, redness, range: −180 (green) to +180 (red); b*, yellowness, range: −180 (blue) to +180 (yellow).

### Analyses of micronutrients of rapeseed oils

**Fig 3:**
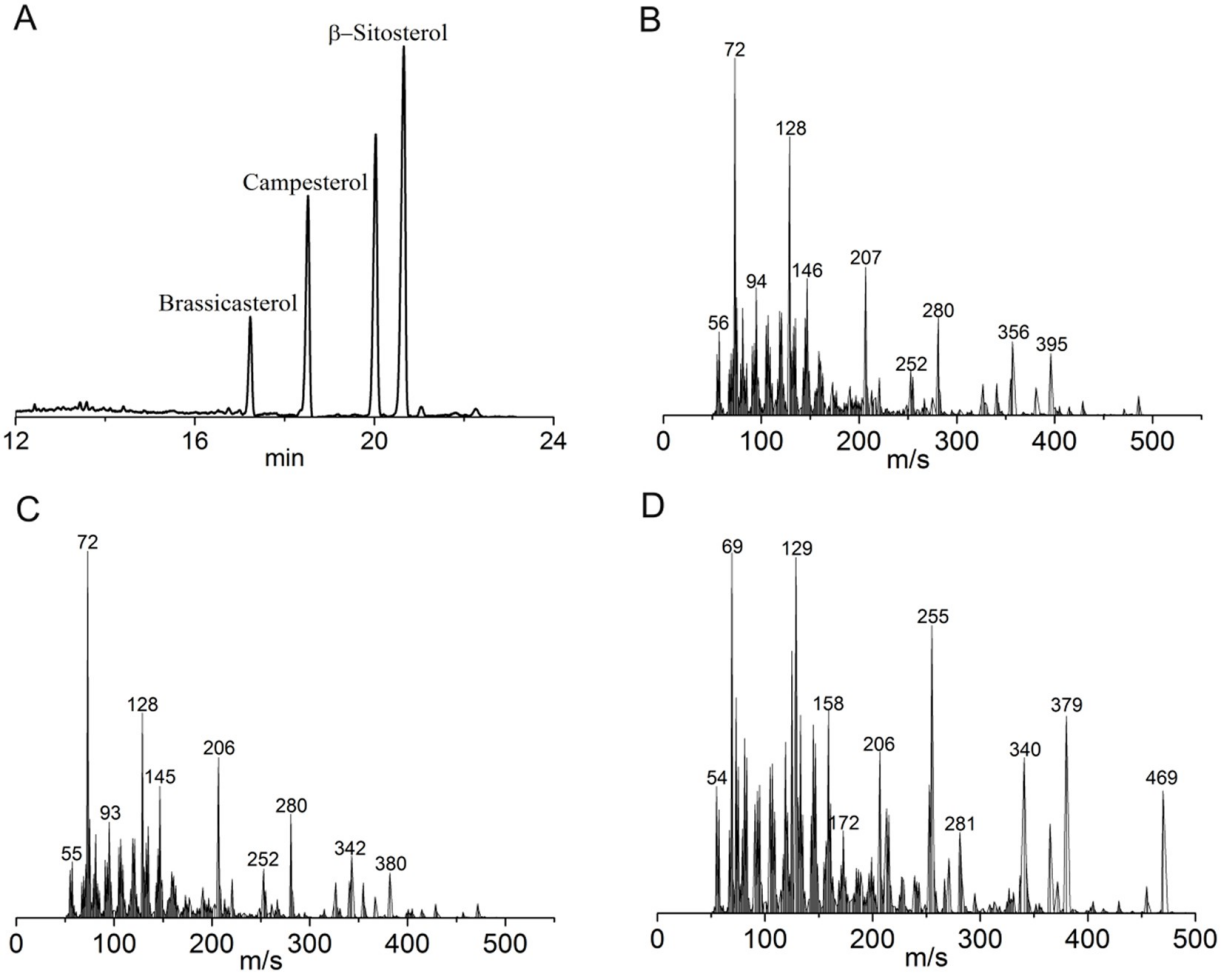
Total ions chromatograph of phytosterols of rapeseed oil analyzed by GC-MS (A); secondary mass spectrum of brassicasterol (B), campesterol (C) and β-sitosterol of rapeseed oil (D).

**Fig 4:**
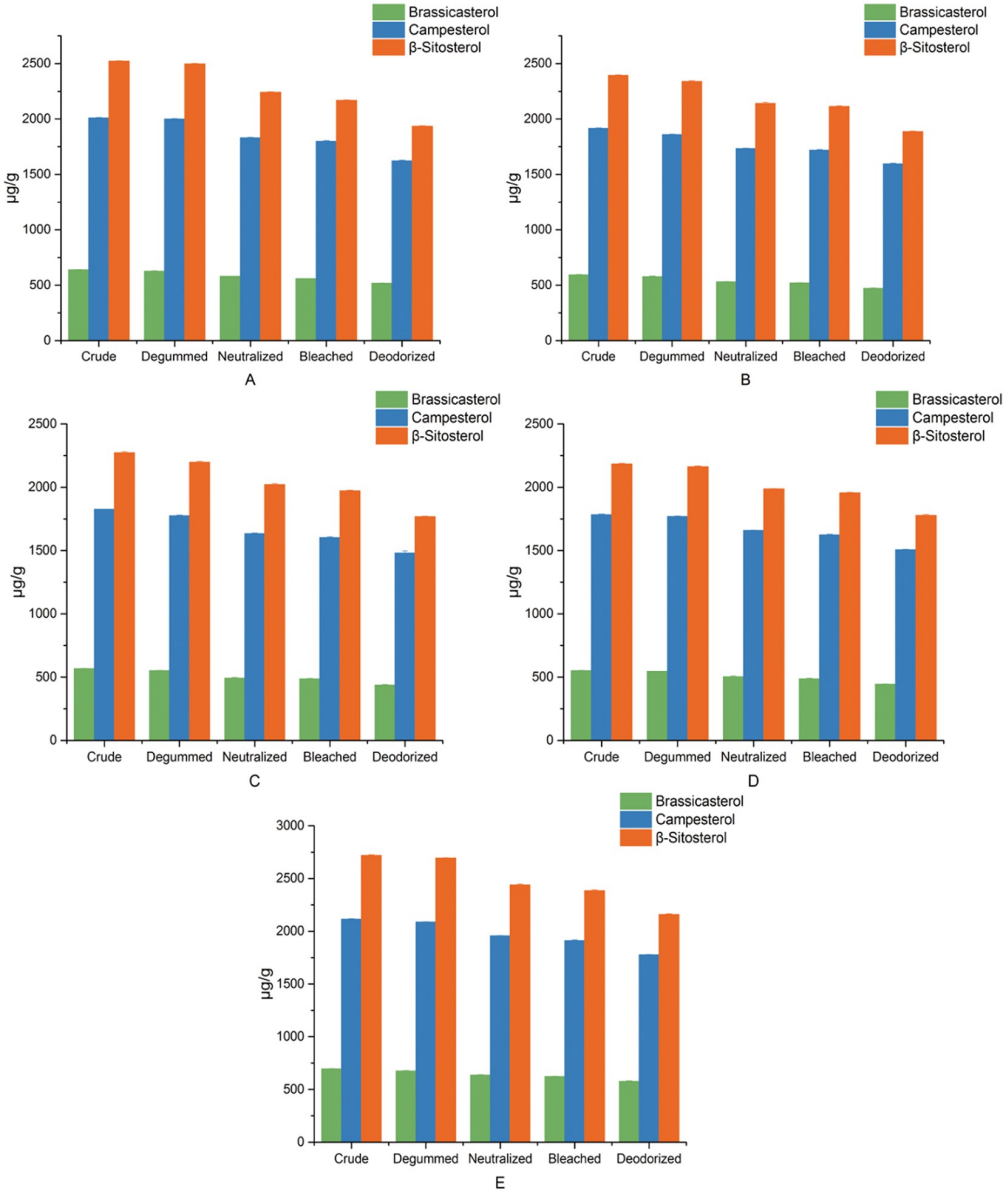
Phytosterols content of oils of number 1 (A), number 2 (B), number 3 (C), number 4 (D), number 5 (E) during every refining step including the degumming, neutralization, bleaching and deodorization.

Phytosterols, constituting a major portion of the unsaponifiable fraction of oils, are encouraged to include in the human diet as their ability to lower blood cholesterol and possess anti-inflammatory, antibacterial, antifungal, anti-ulcerative, and antitumoral activities [26]. The total ions chromatograph and secondary mass spectrum of phytosterols of rapeseed oil were illustrated in Fig 3. Fig 4 showed the compositions and contents of phytosterols in five different kinds of rapeseed oils during the whole refining process. As shown in Fig 4, β-sitosterol was the most abundant phytosterol in all kinds of rapeseed oils, followed by campesterol and brassicasterol. The content of phytosterol in the crude oil samples were from 4518.30–5529.05 μg/g. It has underwent a remarkable decrease after refining, ranging from 3685.75–4512.20μg/g. In this study, the loss rate of brassicasterol, campesterol, β-sitosterol and total phytosterols content of five different kinds of rapeseed oils were similar at the same refining step. Furthermore, the loss rate of neutralization and deodorization were much higher than that of degumming and bleaching for any kind of phytosterol of all oils. The range of loss rate of total phytosterols content of all oils during the degumming, neutralization, bleaching and deodorization were from 0.87 to 3.01%, 7.3 to 9.23%, 1.18 to 2.75% and 8.27 to 9.97%, respectively. A significant reduction in the phytosterols content of all oils during neutralization could be a result of micelle formation (free sterols and soap). In addition, the significant reduction in the phytosterols content of all oils during deodorization may be due to sterols esterification promoted by the deodorization temperature and a reduction in the free sterol content caused by distillation. The reduction of total and individual sterols in rapeseed oils after complete refining process were approximately 5–70% [27], which were agreed with the data of this study.

**Fig 5:**
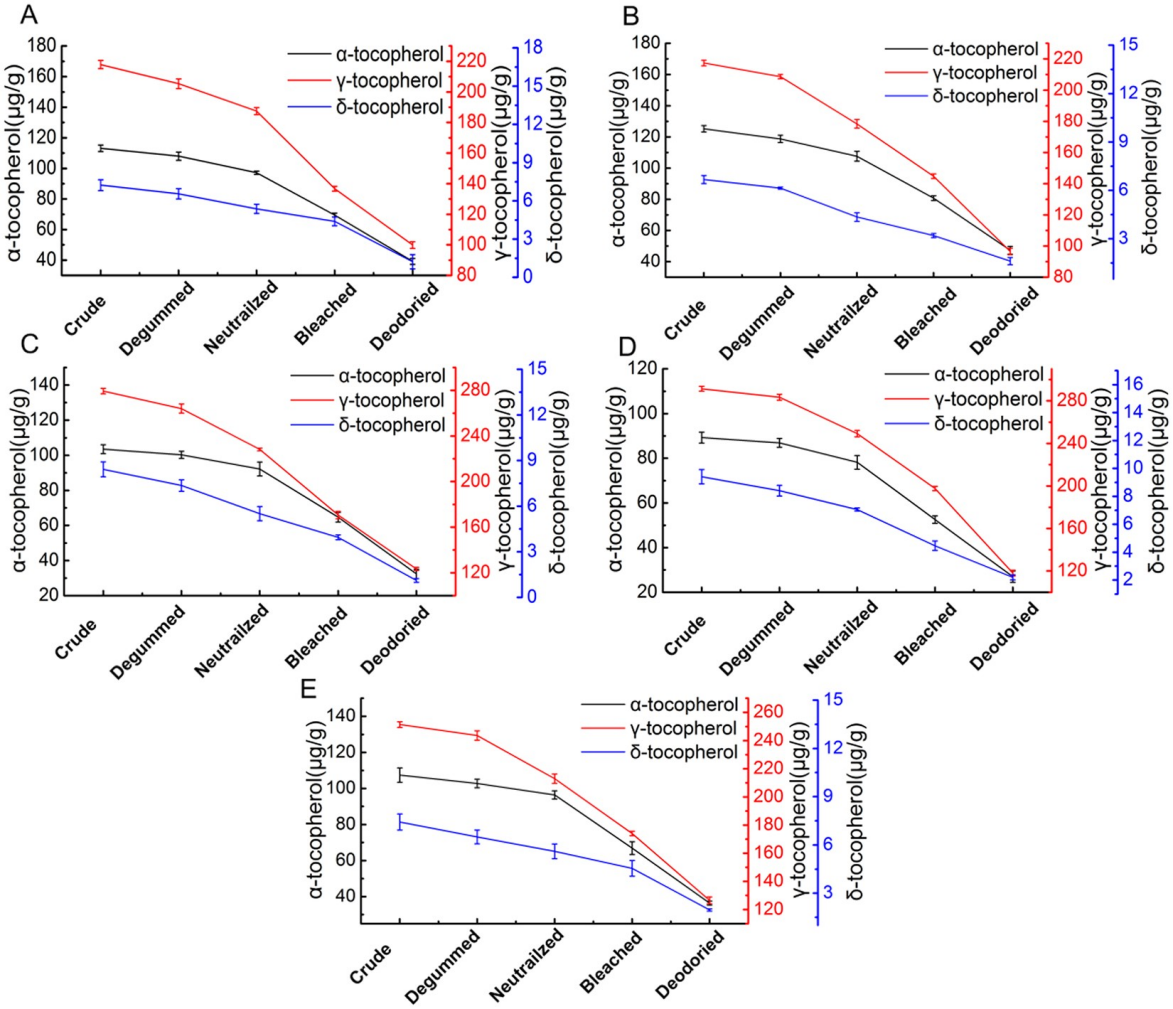
The α-tocopherol, γ-tocopherol and δ-tocopherol contents of oils of number 1 (A), number 2 (B), number 3 (C), number 4 (D), number 5 (E) during every refining step including the degumming, neutralization, bleaching and deodorization.

Tocopherols are a group of fat-soluble antioxidants and could be divided into α-, β-, γ-, and δ- forms [28]. They (α, γ, and δ) are potent natural antioxidants that can inhibit the deterioration of oils during storage and prolong the shelf life of edible oils [29]. Fig 5 showed total and individual tocopherol contents of different rapeseed oil varieties at each step of refining process. The results have shown that γ-tocopherol is the most abundant tocopherol with the concentration from 217.39 μg/g (Fengyou 5103) to 291.32 μg/g (Zhongyou 6766) in crude oils, followed by α-tocopherol, from 89.26 μg/g (Zhongyou 6766) to 125.18μg/g (Fengyou 5103) A significant gradual degradation in tocopherol content was observed during each step of refining process, which was consistent with previous studies [30].

The decrease of α-tocopherol, γ-tocopherol, δ-tocopherol and total tocopherols in different rapeseed oil varieties during the neutralization were from 6.21 to 10.01%, 8.71 to 14.52%, 13.70 to 29.12%, and 9.31 to 12.94%, respectively. The reduction might be due to the instability of tocols in the presence of air and alkali. Previous research [31] reported neutralization could result in the reduction of α-tocopherol (17.4%), followed by δ-tocopherol (16.9%) and γ-tocopherol (12.5%) of sunflower oil, which was different from the data of this study. This was probably owing to different varieties of vegetable oil.

During the bleaching, the range of the loss rate of total tocopherols content of different rapeseed oil varieties was between 21.21 and 27.41%. This reduction could be attributed to the possible adsorption on bleaching clay. Previous research [32] reported bleaching could result in the decrease of about 17.4% of tocopherol content in rapeseed oil process. The loss rate of total tocopherols content of different rapeseed oil varieties (between 32.82 and 42.16%) was found the most during the deodorization, likely owing to the thermal degradation at high temperature or by chemical reaction during deodorization. Previous study reported the greatest losses of total tocopherol content in oils could be induced by physical refining (deodorization), amounting approximately to 20.2–27.1% [32], and the deodorization step would cause 7–90% losses of tocopherols in rapeseed oils [33]. In previous study when the deodorizing operation was performed at 260°C, loss rate of tocopherol was up to 40–50% [34].

**Fig 6:**
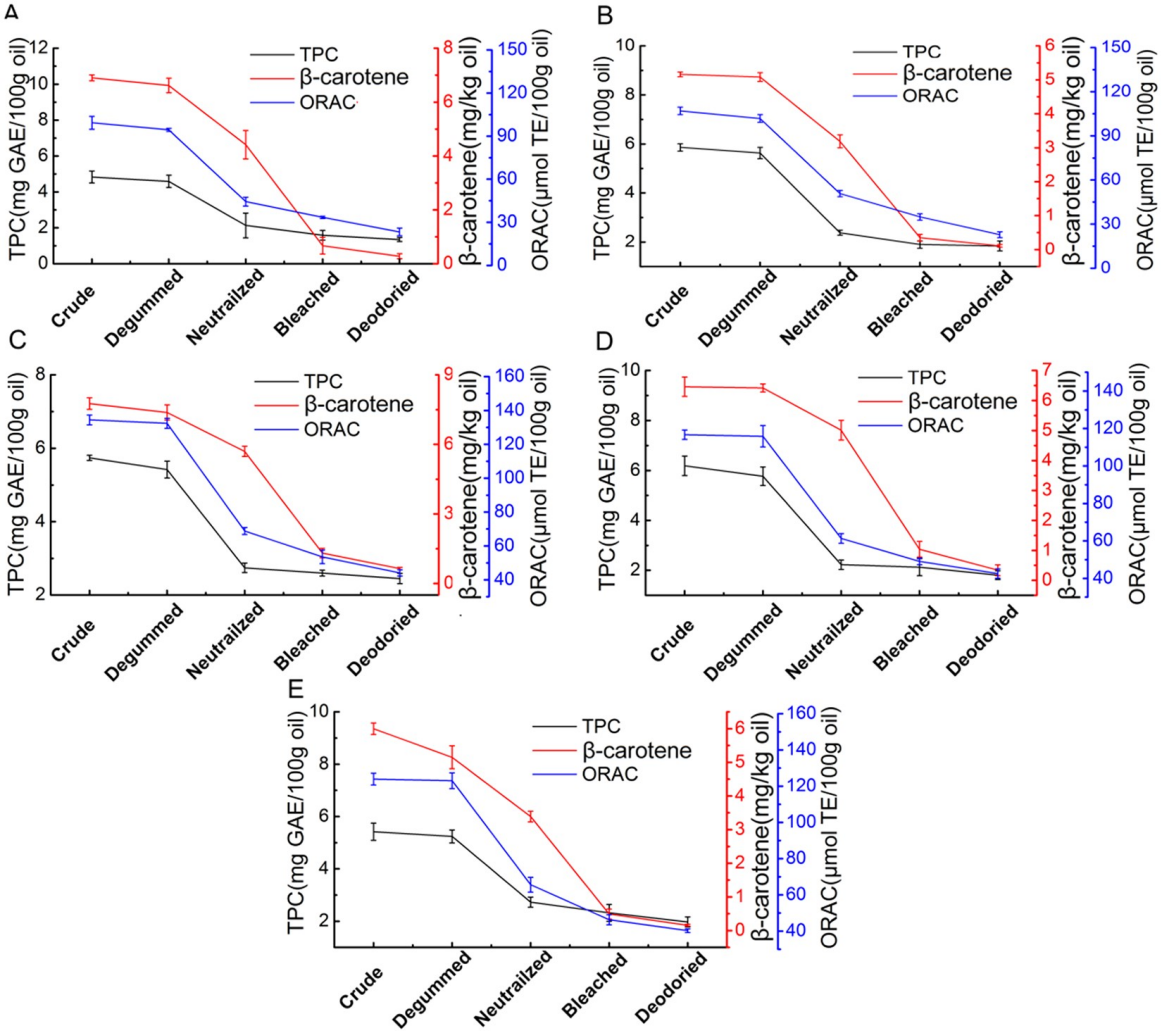
The TPC, β-carotene and ORAC of oils of number 1 (A), number 2 (B), number 3 (C), number 4 (D), number 5 (E) during every refining step including the degumming, neutralization, bleaching and deodorization.

The role of polyphenols in inhibiting the decomposition of hydroperoxides is critical to maintaining food quality by reducing rancidity caused by aldehyde formation [35]. We found that there was a remarkable decrease (4.83–6.19 to 1.35–2.45 mg GAE/100 g oil) after the whole refining process in the TPC of five different kinds of rapeseed oils, as shown in Fig 6. The range of loss of TPC of all oils during neutralization was from 47.93 to 61.19%, which was much higher than degumming (3.21 to 6.61%), bleaching (2.90 to 19.01%) and deodorization (4.81 to 16.62%). This was consistent with previous study [24] in which phenolic compounds would be generally removed from oil after chemical neutralization. Additionally the marked decrease observed in the TPC contents after the neutralization was mainly due to more decrease in the contents of the free fatty acids and oxidized triglyceride monomers than that of the other polar compounds, resulting in the loss of phenolic substances [22].

Fig 6 showed the β-carotene content of five different kinds of rapeseed oils at each refining step. The content of β-carotene of 5 different kinds of crude rapeseed oils was from 5.16 to 7.76 mg/kg oil. In this study, the loss rate of bleaching and deodorization were much higher than degumming and neutralization. β-carotene decreased from 77.05 to 88.91% due to adsorption of bleaching earth. Previous research reported greatest losses of β-carotene were observed when degummed rapeseed oil was bleached with 0.89% w/w acid activated bleaching clay, which caused decrease of β-carotene content by 73% [32]. Deodorization decreased β-carotene from 46.41 to 68.44%, which might be attributed to degradation during high temperature deodorization process. The different loss rate of all oils during deodorization could be owing to the difference of varieties of rapeseed oils.

ORAC assays are often used for comparison to determine the antioxidant capacity of rapeseed oils at various stages of technological process [36]. The ORAC of five different kinds of crude rapeseed oils were from 99.29 to 134.45 μmol TE /100g oil. The range of the loss rate of ORAC of all oils during neutralization (46.93 to 52.94%) was the highest, followed by the bleaching (19.93 to 31.25%), deodorization (12.84 to 34.74%) and degumming (0.81 to 5.09%). The reduction of ORAC during neutralization, bleaching and deodorization may be due to the removal of antioxidants [21], such as phytosterols, tocopherols, polyphenols and carotenoid. The loss of these antioxidants can have a strong negative influence on the oxidative stability of the oil.

**Fig 7:**
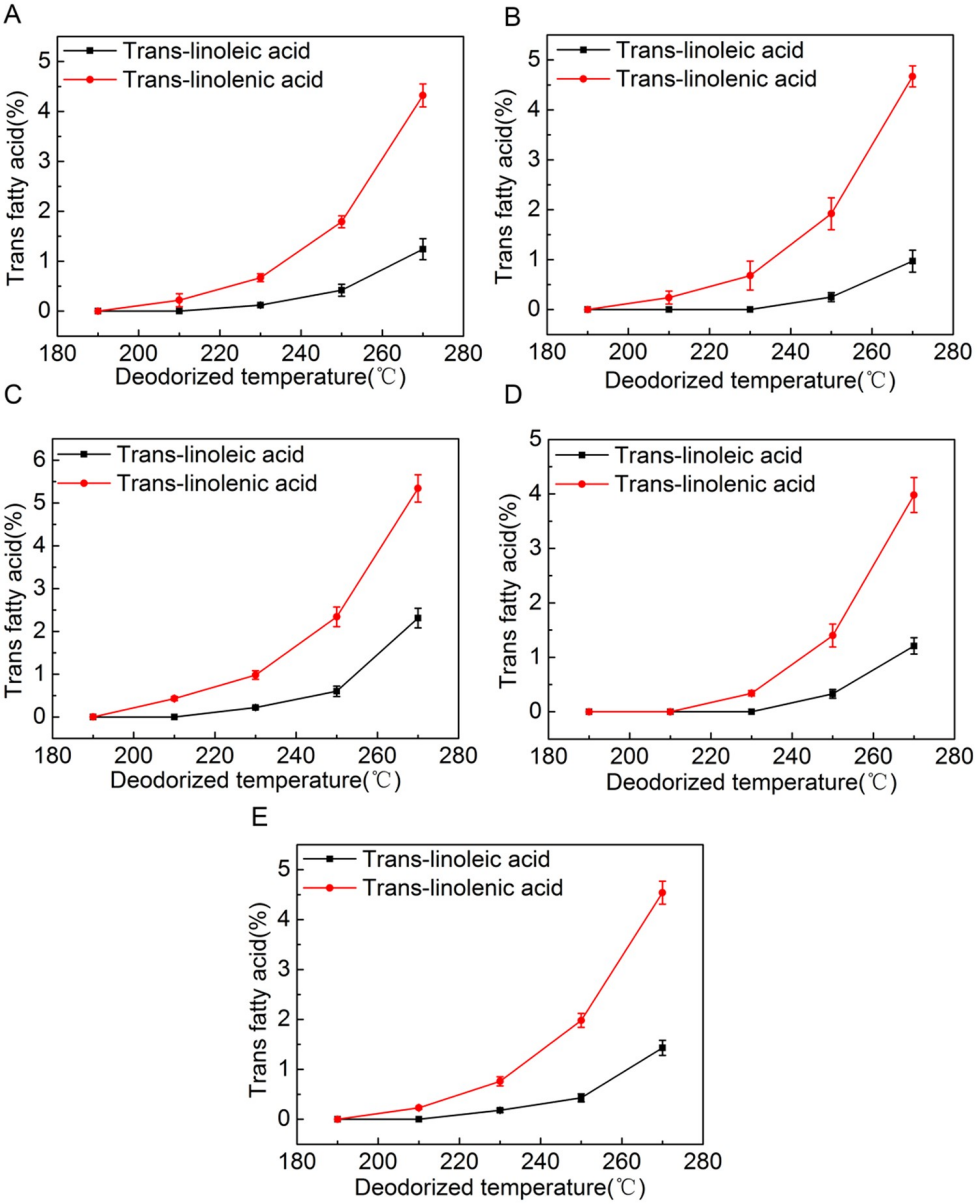
Trans-linoleic and trans-linolenic percentage contents of oils of number 1 (A), number 2 (B), number 3 (C), number 4 (D), number 5 (E) at different deodorized temperature including 190°C, 210°C, 230°C, 250°C and 270°C.

Fig 7 showed that deodorized temperature had affected percentage content of trans-linoleic and trans-linolenic significantly in all kinds of rapeseed oils. We found that there was no trans-linoleic generated at 190°C and 210 °C and no trans-linolenic generated as well at 190°C for each variety of deodorized rapeseed oil. Percentage content of trans fatty acid of deodorized oil of Douyou 8 was higher than that of other four kinds of rapeseed oils which might be due to variety difference of rapeseed oils. In addition, trans-linolenic had the faster growth rate than trans-linoleic acid with the increasing temperature, could be attributed to linolenic containing more carbon double bonds than linoleic, resulting in the much higher possibility of isomerization. Furthermore, the high deodorized temperature (250°C and 270°C) induced the percentage content of trans fatty acid increased much higher than that of low deodorization temperature (190°C, 210°C and 230°C) which indicated that isomerization of double bonds can be promoted under high temperature.

## Conclusions

Considering comprehensively, the neutralization stage and the deodorization stage have the greatest influence on the micronutrients and antioxidant activity of rapeseed oil, followed by the bleaching stage, and the least affected is the degumming stage. A similar trend was observed for the AV, PV and *p*-AV changes of the oils in varieties during refining process, which indicates that there was no significant difference between these chemical properties of rapeseed oil in varieties. Content of phytosterols, tocopherols, TPC, β-carotene and ORAC did not show clear trends for any of the different rapeseed oil varieties, either. This study provides a scientific basis for the necessity of moderate refining of rapeseed oil in actual industrial production.

## Supporting information

S1 Table Acid value of five different kinds of rapeseed oils during the refining process (mgKOH/g). (DOCX)

S2 Table Peroxide value of five different kinds of rapeseed oils during the refining process (mmol/kg oil). (DOCX)

S3 Table p-Anisidine value of five different kinds of rapeseed oils during the refining process. (DOCX)

S4 Table The content of Phytosterols in five different kinds of rapeseed oil (μg/g oil). (DOCX)

S5 Table The contents of tocopherols in five different kinds of oil (μg/g oil). (DOCX)

S6 Table TPC of five different kinds of rapeseed oils (mg GAE/100g oil). (DOCX)

S7 Table The content of β-carotene in five different kinds of rapeseed oil (mg/ 100g oil). (DOCX)

S8 Table The ORAC in five different kinds of rapeseed oil (μmol TE / 100g oil). (DOCX)
