# Supplementary material for: The effect of refining process on the physicochemical properties and micronutrients of rapeseed oils
Source: PLoS One. 2019 Mar 8;14(3):e0212879. doi: 10.1371/journal.pone.0212879 (PMC6407755; doi:10.1371/journal.pone.0212879)
Supplement: S1 Table — (DOCX) [file pone.0212879.s001.docx]

**Table S1**

Acid value of five different kinds of rapeseed oils during the refining process

| Refining process | AV of five different kinds of rapeseed oils ( mgKOH/g) | | | | |
| --- | --- | --- | --- | --- | --- |
|  | Zhongshuang 11 | Fengyou 5103 | Deyou 8 | Zhongyou 6766 | Huyou 4 |
| Crude | 2.14 | 2.53 | 3.64 | 2.75 | 3.06 |
|  | 2.15 | 2.56 | 3.61 | 2.78 | 3.11 |
|  | 2.17 | 2.61 | 3.51 | 2.71 | 3.1 |
| Degummed | 2.06 | 2.43 | 3.57 | 2.65 | 2.95 |
|  | 2.11 | 2.41 | 3.51 | 2.61 | 2.91 |
|  | 2.01 | 2.34 | 3.55 | 2.58 | 2.9 |
| Neutralized | 0.29 | 0.37 | 0.45 | 0.32 | 0.41 |
|  | 0.31 | 0.39 | 0.44 | 0.31 | 0.42 |
|  | 0.27 | 0.41 | 0.45 | 0.34 | 0.43 |
| Bleached | 0.39 | 0.5 | 0.54 | 0.39 | 0.57 |
|  | 0.37 | 0.51 | 0.55 | 0.41 | 0.61 |
|  | 0.41 | 0.44 | 0.61 | 0.46 | 0.61 |
| Deodorized | 0.06 | 0.09 | 0.13 | 0.09 | 0.12 |
|  | 0.07 | 0.11 | 0.08 | 0.1 | 0.09 |
|  | 0.09 | 0.1 | 0.14 | 0.11 | 0.1 |
