# Supplementary material for: The effect of refining process on the physicochemical properties and micronutrients of rapeseed oils
Source: PLoS One. 2019 Mar 8;14(3):e0212879. doi: 10.1371/journal.pone.0212879 (PMC6407755; doi:10.1371/journal.pone.0212879)
Supplement: S2 Table — (DOCX) [file pone.0212879.s002.docx]

**Table S2**

Peroxide value of five different kinds of rapeseed oils during the refining process

| Refining process | PV of five different kinds of rapeseed oils (mmol/kg oil) | | | | |
| --- | --- | --- | --- | --- | --- |
|  | Zhongshuang 11 | Fengyou 5103 | Deyou 8 | Zhongyou 6766 | Huyou 4 |
| Crude | 5.12 | 4.45 | 5.54 | 3.63 | 4.09 |
|  | 5.43 | 4.56 | 5.51 | 3.78 | 4.11 |
|  | 5.44 | 4.76 | 5.15 | 3.29 | 4.21 |
| Degummed | 5.98 | 4.57 | 5.67 | 4.01 | 4.45 |
|  | 5.78 | 4.87 | 5.76 | 4.44 | 4.53 |
|  | 5.46 | 5.09 | 5.78 | 4.21 | 4.67 |
| Neutralized | 6.17 | 5.98 | 6.8 | 5.01 | 5.01 |
|  | 6.76 | 5.56 | 6.78 | 5.38 | 5.11 |
|  | 6.45 | 5.48 | 6.9 | 5.21 | 5.21 |
| Bleached | 4.23 | 3.98 | 5.01 | 3.51 | 3.21 |
|  | 4.22 | 4.01 | 4.76 | 3.11 | 3.33 |
|  | 3.9 | 3.35 | 4.21 | 3.01 | 2.98 |
| Deodorized | 1.34 | 1.56 | 1.51 | 1.2 | 0.89 |
|  | 1.56 | 1.12 | 1.98 | 1.11 | 0.97 |
|  | 1.67 | 1.32 | 1.38 | 0.98 | 1.07 |
