# Supplementary material for: The effect of refining process on the physicochemical properties and micronutrients of rapeseed oils
Source: PLoS One. 2019 Mar 8;14(3):e0212879. doi: 10.1371/journal.pone.0212879 (PMC6407755; doi:10.1371/journal.pone.0212879)
Supplement: S3 Table — (DOCX) [file pone.0212879.s003.docx]

**Table S3**

p-Anisidine value of five different kinds of rapeseed oils during the refining process

| Refining process | PAV of five different kinds of rapeseed oils | | | | |
| --- | --- | --- | --- | --- | --- |
|  | Zhongshuang 11 | Fengyou 5103 | Deyou 8 | Zhongyou 6766 | Huyou 4 |
| Crude | 2.55 | 1.78 | 2.06 | 2.76 | 1.78 |
|  | 2.45 | 1.96 | 2.18 | 2.93 | 1.85 |
|  | 2.69 | 1.9 | 2.08 | 2.59 | 1.71 |
| Degummed | 1.95 | 1.37 | 1.81 | 2.23 | 1.33 |
|  | 2.01 | 1.45 | 1.79 | 2.16 | 1.32 |
|  | 1.85 | 1.39 | 1.71 | 2.05 | 1.25 |
| Neutralized | 2.21 | 1.66 | 1.84 | 2.43 | 1.67 |
|  | 2.36 | 1.64 | 1.79 | 2.28 | 1.55 |
|  | 2.15 | 1.58 | 1.76 | 2.37 | 1.47 |
| Bleached | 1.73 | 1.13 | 1.24 | 1.98 | 0.99 |
|  | 1.79 | 1.01 | 1.22 | 2.08 | 1.01 |
|  | 1.61 | 1.21 | 1.18 | 1.84 | 0.98 |
| Deodorized | 2.03 | 1.46 | 1.34 | 2.35 | 1.62 |
|  | 2.09 | 1.34 | 1.41 | 2.58 | 1.52 |
|  | 1.91 | 1.28 | 1.37 | 2.29 | 1.65 |
