# Supplementary material for: The effect of refining process on the physicochemical properties and micronutrients of rapeseed oils
Source: PLoS One. 2019 Mar 8;14(3):e0212879. doi: 10.1371/journal.pone.0212879 (PMC6407755; doi:10.1371/journal.pone.0212879)
Supplement: S4 Table — (DOCX) [file pone.0212879.s004.docx]

**Table S4**

The content of Phytosterols in five different kinds of rapeseed oil

| The content of Phytosterols in rapeseed oil of Zhongshuang 11 (μg/g oil) | | | | |
| --- | --- | --- | --- | --- |
| Refining process | Brassicasterol | Campesterol | β-Sitosterol | Total phytosterols |
| Crude | 127.13 | 401.14 | 504.47 | 1032.74 |
|  | 128.14 | 402.22 | 503.29 | 1033.65 |
|  | 127.98 | 402.98 | 505.33 | 1036.29 |
| Degummed | 124.82 | 399.6 | 499.09 | 1023.51 |
|  | 125.27 | 399.98 | 499.03 | 1024.28 |
|  | 126.14 | 401.22 | 500.29 | 1027.65 |
| Neutralized | 115.62 | 366.23 | 449.36 | 931.21 |
|  | 115.67 | 365.29 | 448.29 | 929.25 |
|  | 116.27 | 367.29 | 447.38 | 930.94 |
| Bleached | 111.23 | 359.27 | 432.91 | 903.41 |
|  | 112.23 | 358.29 | 433.09 | 903.61 |
|  | 111.98 | 361.22 | 434.51 | 907.71 |
| Deodorized | 103.28 | 325.81 | 385.48 | 814.57 |
|  | 104.23 | 324.29 | 387.29 | 815.81 |
|  | 102.72 | 323.48 | 387.28 | 813.48 |

| The content of Phytosterols in rapeseed oil of Fengyou 5103 (μg/g oil) | | | | |
| --- | --- | --- | --- | --- |
| Refining process | Brassicasterol | Campesterol | β-Sitosterol | Total phytosterols |
| Crude | 119.66 | 382.39 | 477.92 | 979.97 |
|  | 118.09 | 383.98 | 478.98 | 981.05 |
|  | 117.87 | 382.76 | 479.09 | 979.72 |
| Degummed | 115.24 | 371.74 | 468.83 | 955.81 |
|  | 114.29 | 372.98 | 467.9 | 955.17 |
|  | 116.76 | 370.98 | 465.77 | 953.51 |
| Neutralized | 106.54 | 347.21 | 429.78 | 883.53 |
|  | 105.25 | 345.87 | 428.98 | 880.1 |
|  | 106.38 | 346.98 | 425.76 | 879.12 |
| Bleached | 103.92 | 342.89 | 423.76 | 870.57 |
|  | 104.9 | 342.88 | 421.09 | 868.87 |
|  | 103.87 | 345.28 | 422.98 | 872.13 |
| Deodorized | 93.22 | 319.25 | 378.38 | 790.85 |
|  | 94.23 | 318.27 | 377.09 | 789.59 |
|  | 94.98 | 320.66 | 376.38 | 792.02 |

| The content of Phytosterols in rapeseed oil of Deyou 8 (μg/g oil) | | | | |
| --- | --- | --- | --- | --- |
| Refining process | Brassicasterol | Campesterol | β-Sitosterol | Total phytosterols |
| Crude | 119.66 | 382.39 | 477.92 | 979.97 |
|  | 118.09 | 383.98 | 478.98 | 981.05 |
|  | 117.87 | 382.76 | 479.09 | 979.72 |
| Degummed | 115.24 | 371.74 | 468.83 | 955.81 |
|  | 114.29 | 372.98 | 467.9 | 955.17 |
|  | 116.76 | 370.98 | 465.77 | 953.51 |
| Neutralized | 106.54 | 347.21 | 429.78 | 883.53 |
|  | 105.25 | 345.87 | 428.98 | 880.1 |
|  | 106.38 | 346.98 | 425.76 | 879.12 |
| Bleached | 103.92 | 342.89 | 423.76 | 870.57 |
|  | 104.9 | 342.88 | 421.09 | 868.87 |
|  | 103.87 | 345.28 | 422.98 | 872.13 |
| Deodorized | 93.22 | 319.25 | 378.38 | 790.85 |
|  | 94.23 | 318.27 | 377.09 | 789.59 |
|  | 94.98 | 320.66 | 376.38 | 792.02 |

| The content of Phytosterols in rapeseed oil of Zhongyou 6766 (μg/g oil) | | | | |
| --- | --- | --- | --- | --- |
| Refining process | Brassicasterol | Campesterol | β-Sitosterol | Total phytosterols |
| Crude | 109.82 | 356.28 | 436.49 | 902.59 |
|  | 110.99 | 357.87 | 435.55 | 904.41 |
|  | 109.78 | 356.22 | 437.98 | 903.98 |
| Degummed | 108.92 | 353.27 | 431.11 | 893.3 |
|  | 109.08 | 354.98 | 432.98 | 897.04 |
|  | 108.87 | 353.1 | 433.23 | 895.2 |
| Neutralized | 100.27 | 331.21 | 397.78 | 829.26 |
|  | 102.22 | 332.09 | 397.89 | 832.2 |
|  | 99.09 | 332.09 | 396.87 | 828.05 |
| Bleached | 97.23 | 324.27 | 391.25 | 812.75 |
|  | 98.22 | 324.09 | 390.23 | 812.54 |
|  | 96.2 | 325.98 | 392.09 | 814.27 |
| Deodorized | 88.98 | 301.23 | 354.21 | 744.42 |
|  | 89.2 | 302.22 | 356.99 | 748.41 |
|  | 87.99 | 300.22 | 355.34 | 743.55 |

| The content of Phytosterols in rapeseed oil of Huyou 4(μg/g oil) | | | | |
| --- | --- | --- | --- | --- |
| Refining process | Brassicasterol | Campesterol | β-Sitosterol | Total phytosterols |
| Crude | 138.63 | 422.45 | 542.56 | 1103.64 |
|  | 138.98 | 421.98 | 543.99 | 1104.95 |
|  | 139.77 | 423.99 | 545.09 | 1108.85 |
| Degummed | 135.35 | 417.78 | 539.28 | 1092.41 |
|  | 135.98 | 416.9 | 538.98 | 1091.86 |
|  | 134.09 | 418.28 | 538.01 | 1090.38 |
| Neutralized | 127.09 | 391.16 | 489.53 | 1007.78 |
|  | 128.3 | 392.22 | 487.87 | 1008.39 |
|  | 126.13 | 391.09 | 485.9 | 1003.12 |
| Bleached | 125.2 | 383.93 | 478.73 | 987.86 |
|  | 124.21 | 382.09 | 476.98 | 983.28 |
|  | 123.98 | 381 | 475.28 | 980.26 |
| Deodorized | 116.27 | 354.98 | 431.99 | 903.24 |
|  | 115.98 | 356.09 | 432.98 | 905.05 |
|  | 113.87 | 355.28 | 429.87 | 899.02 |
