# Supplementary material for: The effect of refining process on the physicochemical properties and micronutrients of rapeseed oils
Source: PLoS One. 2019 Mar 8;14(3):e0212879. doi: 10.1371/journal.pone.0212879 (PMC6407755; doi:10.1371/journal.pone.0212879)
Supplement: S5 Table — (DOCX) [file pone.0212879.s005.docx]

**Table S5**

The contents of tocopherols in five different kinds of oil

| The content of tocopherols in rapeseed oil of Zhongshuang 11(μg/g oil) | | | | |
| --- | --- | --- | --- | --- |
| Refining process | α-tocopherol | λ-tocopherol | δ-tocopherol | total tocopherol |
| Crude | 112.98 | 218.53 | 6.99 | 338.5 |
|  | 113.22 | 217.9 | 7.01 | 338.13 |
|  | 113.23 | 217.22 | 7.72 | 338.17 |
| Degummed | 108.29 | 205.66 | 6.87 | 320.82 |
|  | 107.09 | 204.12 | 6.09 | 317.3 |
|  | 108.32 | 206.32 | 6.71 | 321.35 |
| Neutralized | 98.23 | 188.15 | 5.78 | 292.16 |
|  | 97.21 | 188.21 | 5.09 | 290.51 |
|  | 96.12 | 186.02 | 5.23 | 287.37 |
| Bleached | 70.67 | 135.13 | 4.77 | 210.57 |
|  | 69.29 | 136.98 | 4.09 | 210.36 |
|  | 68.2 | 138.09 | 4.31 | 210.6 |
| Deodorized | 38.41 | 99.25 | 0.91 | 138.57 |
|  | 39.02 | 98.12 | 0.88 | 138.02 |
|  | 40.23 | 102.34 | 1.87 | 144.44 |

| The content of Tocopherols in rapeseed oil of Fengyou 5103(μg/g oil) | | | | |
| --- | --- | --- | --- | --- |
| Refining process | α-tocopherol | λ-tocopherol | δ-tocopherol | total tocopherol |
| Crude | 126.21 | 216.85 | 6.4 | 349.46 |
|  | 124.09 | 216.98 | 6.9 | 347.97 |
|  | 125.23 | 218.33 | 6.72 | 350.28 |
| Degummed | 119.97 | 208.97 | 6.21 | 335.15 |
|  | 118.87 | 209.89 | 6.09 | 334.85 |
|  | 117.32 | 207.22 | 6.11 | 330.65 |
| Neutralized | 108.51 | 179.26 | 4.62 | 292.39 |
|  | 108.09 | 178.09 | 4.09 | 290.27 |
|  | 106.32 | 177.79 | 4.33 | 288.44 |
| Bleached | 81.02 | 143.23 | 3.07 | 227.32 |
|  | 82.09 | 144.98 | 3.2 | 230.27 |
|  | 79.09 | 146.23 | 3.33 | 228.65 |
| Deodorized | 45.87 | 94.23 | 1.49 | 141.59 |
|  | 47.09 | 97.2 | 1.5 | 145.79 |
|  | 48.87 | 98.23 | 1.87 | 148.97 |

| The content of Tocopherols in rapeseed oil of Deyou 8(μg/g oil) | | | | |
| --- | --- | --- | --- | --- |
| Refining process | α-tocopherol | λ-tocopherol | δ-tocopherol | total tocopherol |
| Crude | 103.56 | 279.36 | 8.19 | 391.11 |
|  | 101.98 | 277.09 | 8.09 | 387.16 |
|  | 104.99 | 281.65 | 8.98 | 395.62 |
| Degummed | 101.23 | 264.04 | 7.77 | 373.04 |
|  | 100.32 | 265.09 | 7.29 | 372.7 |
|  | 99.21 | 263.11 | 7.01 | 369.33 |
| Neutralized | 93.16 | 229.47 | 5.68 | 328.31 |
|  | 92.1 | 227.09 | 5.87 | 325.06 |
|  | 91.32 | 228.12 | 4.98 | 324.42 |
| Bleached | 65.22 | 169.68 | 4.09 | 238.99 |
|  | 65.09 | 172.1 | 3.98 | 241.17 |
|  | 63.87 | 170.23 | 3.78 | 237.88 |
| Deodorized | 31.87 | 121.89 | 1 | 154.76 |
|  | 30.99 | 123.98 | 1.09 | 156.06 |
|  | 34.21 | 124.51 | 1.23 | 159.95 |

| The content of Tocopherols in rapeseed oil of Zhongyou 6766(μg/g oil) | | | | |
| --- | --- | --- | --- | --- |
| Refining process | α-tocopherol | λ-tocopherol | δ-tocopherol | total tocopherol |
| Crude | 89.78 | 293.59 | 9.2 | 392.57 |
|  | 88.98 | 289.09 | 9.98 | 388.05 |
|  | 89.01 | 291.27 | 9.01 | 389.29 |
| Degummed | 86.59 | 282.68 | 8.84 | 378.11 |
|  | 87.98 | 284.23 | 8.27 | 380.48 |
|  | 86.05 | 283.09 | 8.12 | 377.26 |
| Neutralized | 79.3 | 249.76 | 7.03 | 336.09 |
|  | 78.09 | 250.87 | 7.19 | 336.15 |
|  | 77.12 | 246.98 | 6.97 | 331.07 |
| Bleached | 51.82 | 195.37 | 4.75 | 251.94 |
|  | 53.09 | 198.09 | 4.09 | 255.27 |
|  | 52.78 | 199.32 | 4.55 | 256.65 |
| Deodorized | 25.45 | 115.37 | 2.01 | 142.83 |
|  | 26.98 | 118.23 | 2.22 | 147.43 |
|  | 28.57 | 120.66 | 2.36 | 151.59 |

| The content of Tocopherols in rapeseed oil of Huyou 4(μg/g oil) | | | | |
| --- | --- | --- | --- | --- |
| Refining process | α-tocopherol | λ-tocopherol | δ-tocopherol | total tocopherol |
| Crude | 106.35 | 250.21 | 7.29 | 363.85 |
|  | 107.77 | 252.09 | 7.99 | 367.85 |
|  | 108.23 | 251.87 | 7.01 | 367.11 |
| Degummed | 102.34 | 244.71 | 6.99 | 354.04 |
|  | 102.99 | 243.98 | 6.23 | 353.2 |
|  | 103.02 | 242.09 | 6.28 | 351.39 |
| Neutralized | 95.21 | 211.68 | 5.73 | 312.62 |
|  | 96.21 | 212.9 | 5.09 | 314.2 |
|  | 97.78 | 214.21 | 5.98 | 317.97 |
| Bleached | 65.33 | 173.2 | 4.65 | 243.18 |
|  | 67.09 | 175.82 | 4.98 | 247.89 |
|  | 68.32 | 172.98 | 4.01 | 245.31 |
| Deodorized | 35.09 | 124.37 | 1.89 | 161.35 |
|  | 36.99 | 129.09 | 1.96 | 168.04 |
|  | 37.09 | 126.23 | 2.02 | 165.34 |
