# Supplementary material for: The effect of refining process on the physicochemical properties and micronutrients of rapeseed oils
Source: PLoS One. 2019 Mar 8;14(3):e0212879. doi: 10.1371/journal.pone.0212879 (PMC6407755; doi:10.1371/journal.pone.0212879)
Supplement: S6 Table — (DOCX) [file pone.0212879.s006.docx]

**Table S6**

TPC of five different kinds of rapeseed oils ( mg GAE/100g oil)

| Refining process | TPC of five different kinds of rapeseed oils ( mg GAE/100g oil) | | | | |
| --- | --- | --- | --- | --- | --- |
|  | Zhongshuang 11 | Fengyou 5103 | Deyou 8 | Zhongyou 6766 | Huyou 4 |
| Crude | 4.83 | 4.99 | 2.13 | 1.59 | 1.35 |
|  | 4.5 | 4.23 | 2.82 | 1.52 | 1.21 |
|  | 5.16 | 4.55 | 1.44 | 1.47 | 1.38 |
| Degummed | 5.86 | 5.63 | 2.38 | 1.92 | 1.64 |
|  | 6.31 | 5.46 | 2.57 | 1.74 | 1.5 |
|  | 5.71 | 5.8 | 2.19 | 1.75 | 1.62 |
| Neutralized | 5.44 | 5.42 | 2.74 | 2.6 | 2.45 |
|  | 5.67 | 5.65 | 2.87 | 2.37 | 2.12 |
|  | 5.81 | 5.19 | 2.61 | 2.56 | 2.17 |
| Bleached | 5.19 | 5.17 | 2.23 | 2.13 | 1.81 |
|  | 6.8 | 6.74 | 2.36 | 2.18 | 1.62 |
|  | 6.58 | 5.4 | 2.08 | 2.22 | 1.71 |
| Deodorized | 5.42 | 5.24 | 2.73 | 2.32 | 1.97 |
|  | 5.79 | 5.99 | 2.92 | 2.37 | 2.13 |
|  | 5.05 | 4.49 | 2.54 | 2.36 | 2.06 |
