# Supplementary material for: The effect of refining process on the physicochemical properties and micronutrients of rapeseed oils
Source: PLoS One. 2019 Mar 8;14(3):e0212879. doi: 10.1371/journal.pone.0212879 (PMC6407755; doi:10.1371/journal.pone.0212879)
Supplement: S7 Table — (DOCX) [file pone.0212879.s007.docx]

**Table S7**

The content of β-carotene in five different kinds of rapeseed oil (mg/ 100g oil)

| Refining process | The content of β-carotene in five different kinds of rapeseed oil (mg/ 100g oil) | | | | |
| --- | --- | --- | --- | --- | --- |
|  | Zhongshuang 11 | Fengyou 5103 | Deyou 8 | Zhongyou 6766 | Huyou 4 |
| Crude | 6.84 | 5.2 | 7.91 | 6.34 | 5.82 |
|  | 7.03 | 5.21 | 7.89 | 6.83 | 6.04 |
|  | 6.83 | 5.08 | 7.47 | 6.22 | 6.15 |
| Degummed | 6.92 | 4.94 | 7.14 | 6.3 | 5.47 |
|  | 6.39 | 5.2 | 7.24 | 6.56 | 4.8 |
|  | 6.55 | 5.1 | 7.76 | 6.41 | 5.19 |
| Neutralized | 3.81 | 3.07 | 5.95 | 5.24 | 3.22 |
|  | 4.68 | 3.41 | 5.54 | 4.63 | 3.41 |
|  | 4.77 | 3.1 | 5.62 | 5.15 | 3.54 |
| Bleached | 0.54 | 0.26 | 1.4 | 1.3 | 0.59 |
|  | 0.44 | 0.35 | 1.08 | 0.78 | 0.55 |
|  | 1.02 | 0.45 | 1.45 | 1.05 | 0.32 |
| Deodorized | 0.21 | 0.16 | 0.64 | 0.55 | 0.19 |
|  | 0.38 | 0.09 | 0.59 | 0.23 | 0.17 |
|  | 0.26 | 0.08 | 0.69 | 0.24 | 0.13 |
