# Supplementary material for: The effect of refining process on the physicochemical properties and micronutrients of rapeseed oils
Source: PLoS One. 2019 Mar 8;14(3):e0212879. doi: 10.1371/journal.pone.0212879 (PMC6407755; doi:10.1371/journal.pone.0212879)
Supplement: S8 Table — (DOCX) [file pone.0212879.s008.docx]

**Table S8**

The ORAC in five different kinds of rapeseed oil (μmol TE / 100g oil)

| Refining process | The ORAC in five different kinds of rapeseed oil (μmol TE / 100g oil) | | | | |
| --- | --- | --- | --- | --- | --- |
|  | Zhongshuang 11 | Fengyou 5103 | Deyou 8 | Zhongyou 6766 | Huyou 4 |
| Crude | 97.34 | 104.35 | 89.09 | 113.42 | 127.43 |
|  | 89.09 | 107.32 | 87.32 | 115.34 | 120.98 |
|  | 96.43 | 109.34 | 83.23 | 118.34 | 123.23 |
| Degummed | 100.32 | 102.29 | 81.23 | 110.32 | 119.09 |
|  | 98.23 | 99.09 | 83.32 | 119.2 | 125.23 |
|  | 99.43 | 104.23 | 88.9 | 121.09 | 127.43 |
| Neutralized | 45.23 | 53.2 | 44.23 | 62.31 | 70.21 |
|  | 46.89 | 50.21 | 46.23 | 63.21 | 62.32 |
|  | 40.98 | 49.03 | 48.32 | 58.43 | 64.32 |
| Bleached | 34.24 | 34.23 | 34.23 | 51.09 | 48.34 |
|  | 33.09 | 37.32 | 38.09 | 48.09 | 47.23 |
|  | 32.89 | 33.12 | 29.09 | 47.99 | 43.12 |
| Deodorized | 35.34 | 32.1 | 31.22 | 45.23 | 50.23 |
|  | 34.21 | 34.98 | 29.06 | 42.13 | 51.21 |
|  | 30.32 | 31.29 | 28.09 | 40.32 | 49.23 |
